# Surgery of the Vermiform Appendix

**Published:** 1895-05-25

**Authors:** 


### PROGRESS IN SURGERY.

### SURGERY OF THE VERMIFORM APPENDIX.

*(Continued from page 100.)*

**The Incision in the Abdominal Wall** in cases of appendicitis often leads to hernia, and to prevent this McBurney[8] recommends the following method: The incision in the skin is an oblique one, about four inches long. It crosses a line drawn from the anterior iliac spine to the umbilicus nearly at right angles, about one inch from the iliac spine, and is so situated that its upper third lies above that line. The section of the external oblique muscle and aponeurosis should correspond, great care being taken to separate these tissues in the same line, not cutting any fibres across. When the edges of the wound in the external oblique are strongly retracted, a considerable extent of the internal oblique muscle is seen, the fibres of which cross somewhat obliquely the opening formed by these retractors. With a blunt instrument, such as the handle of a knife, the fibres of the internal oblique and transversalis muscles can now be separated, without cutting more than an occasional fibre, in a line parallel with their course, that is, nearly at right angles to the incision in the external oblique aponeurosis. Blunt retractors should now be introduced into this in turn and the edges separated. The transversalis fascia is thus well exposed and is then divided in the same line. Last of all, the section of the peritoneum is made. When the operation is completed it will be seen that the gridiron arrangement of the muscular and tendinous fibres, to which the abdominal wall largely owes its strength, is restored almost as completely as if no operation had been done. The operation is not easy, and should not be attempted by those who are unfamiliar with operations upon the appendix, and two extra assistants will be occupied part of the time with retractors. It is not suitable for cases accompanied by suppuration about the appendix, which require to be treated by extensive packing with gauze; nor for non-suppurative cases which require during operation a large intra-abdominal dissection.

**Recurrent Appendicitis.**—Robson[9] reports eight cases operated upon between the attacks. This period is chosen (*a*) because the patient is likely to be in the best possible condition; (*b*) because there is less likelihood of an extensive collection of inflammatory products in or near the appendix, and therefore less danger of soiling the general peritoneal cavity; (*c*) an operation in the quiescent period rarely requires drainage, and therefore there is less risk of a subsequent hernia; (*d*) the appendix can be dealt with in a more satisfactory manner than when it is acutely inflamed and hidden by greatly distended intestines. Although the author does not agree with those who argue that every case of recurrent appendicitis should be operated upon, he believes that operation should be resorted to as soon as it becomes evident that there is a probability of the attack being repeated, especially as the operation is one that can be undertaken in the quiescent period with every prospect of immediate and ultimate success.

[8] Annals of Surgery, July, 1894, p. 38. [9] Therap. Gazette, Sept. 15th, 1894.

